# Effects of Grazing Hour in a Part-Time Grazing System for Dairy Cows on Herbage Intake Assessed by Acoustic Detection of Prehension Events

**DOI:** 10.3390/ani16152373

**Published:** 2026-08-03

**Authors:** Salvatore Bognanno, Filippo Gimmillaro, Marcella Avondo

**Affiliations:** Department of Agriculture, Feeding and Environment, University of Catania, 95123 Catania, Italy; salvatorebgn@gmail.com (S.B.); filippogimmillaro@live.it (F.G.)

**Keywords:** grazing hour, dairy cows, herbage intake, herbage mass, acoustic monitoring

## Abstract

A study was carried out to evaluate the within-session grazing dynamics on bite mass, number of bites and herbage intake in dairy cows, combining acoustic detection of prehension events with hand-plucking estimates of bite mass. Nine lactating cows were managed under a part-time grazing system, with animals allowed access to pasture for 5 h per day. Measurements were conducted on four sampling days at 15-day intervals over a 2-month period. Measurements were taken at pasture entry and after 1, 2, 3, and 4 h of grazing. The number of prehensions during grazing was automatically detected from sound recordings using a custom-developed software tool. Hourly herbage intake was estimated as the product of hourly prehension number and estimated bite mass. Daily intake was also independently calculated from the difference between pre- and post-grazing herbage biomass. All measurements were aggregated at the hourly level to ensure temporal alignment among pasture characteristics, bite mass, and acoustic data. Intake was also independently estimated from biomass disappearance. A significant decrease in biomass (*p* = 0.025) and herbage height (*p* = 0.008) was observed during the grazing session. Bite mass was significantly affected by grazing hour (*p* = 0.008), showing a progressive decrease over time. Grazing hour also affected (*p* < 0.001) the number of prehensions and herbage intake both of which decreased progressively from the beginning to the end of the grazing session. The temporal decline in intake components may reflect both the progressive reduction in available herbage and a decreasing motivation to graze as satiety developed. Mean daily intake estimated from biomass disappearance and from the acoustic approach combined with hand-plucking was 10.71 ± 1.84 and 10.50 ± 2.25 kg DM/cow, respectively. These results suggest that the combined use of acoustic detection of prehensions and measured bite mass may provide a reliable and relatively easy-to-apply method for estimating individual herbage intake in short-duration grazing systems.

## Simple Summary

Short-duration grazing systems are widely used in semi-extensive dairy farming, particularly in marginal hilly and mountain areas. In these systems, pasture often represents the main feed resource, and its use should be carefully managed to optimize animal performance. Feeding behaviour is a key aspect to monitor, as it can provide useful information for adjusting grazing management and feed supplementation. Among behavioural variables, individual herbage intake is particularly important because it is highly sensitive to changes in pasture availability and structure, but it is also difficult to estimate under field conditions. During a grazing session, pasture depletion progressively modifies herbage availability and characteristics, with possible effects on animal ingestive behaviour. This study evaluated behavioural responses to these within-session changes by using an approach based on the acoustic detection of prehensions combined with hand-plucking measurements of bite mass, which mimics the selective grazing behaviour of animals. The proposed approach showed good agreement and potential for estimating individual herbage intake and its variation over time during grazing, while remaining relatively easy to apply under field conditions. This may represent a useful tool for improving pasture and feeding management in semi-extensive dairy systems.

## 1. Introduction

Short-duration grazing systems are widely used in semi-extensive dairy farming, particularly in marginal hilly and mountain areas. In these systems, pasture often represents the main feed resource, and its use should be carefully managed to optimize animal performance. These systems are especially common in dairy production systems based on twice-daily milking. A key feature of short-duration grazing systems is that, unlike continuous 24-h grazing, animals have access to pasture for a limited period of time, within which they must achieve adequate daily herbage intake, often relying exclusively on pasture as their sole feed source.

The detection of grazing behaviour in ruminants is traditionally carried out through direct observations following the animals as they graze, or indirectly, through recording systems, when the animals are confined to limited grazing spaces. However, monitoring feeding behaviour under free-grazing conditions remains challenging, particularly in large and heterogeneous pastures. In such conditions, the availability of automatic detection tools becomes essential. Several automated approaches have been developed, including GPS-based tracking systems, accelerometers, and noseband sensors capable of detecting jaw movements [1,2]. These systems allow the identification of general behavioural states such as grazing, ruminating, or resting. However, the accurate quantification of herbage intake remains difficult. According to the classical definition, herbage intake results from the interaction between eating time, bite rate, and bite mass [3]. Among these components, bite mass is generally considered the most difficult to quantify under grazing conditions, due to the high variability of sward structure and the difficulty of direct measurement in free-ranging animals.

Bite mass can be estimated indirectly by calculating herbage intake from the difference between pre- and post-grazing biomass and dividing the estimated intake by the number of prehensions automatically detected and the number of animals grazing the considered area [4]. However, estimates based on herbage disappearance are often imprecise and do not allow for individual-level assessment. Alternative approaches for estimating herbage mass have been comprehensively reviewed by Susruthan et al. (2025) [5].

Acoustic and video recording systems represent promising tools for the detailed analysis of ingestive behaviour, as they allow the identification of grazing activities based on characteristic acoustic and visual patterns. In particular, the detection of sounds associated with herbage prehension, and chewing has been proposed as a valid alternative to traditional methods, given its sensitivity in identifying feeding events [6]. Sound recording can effectively characterize feeding behaviour, as bite prehension, chewing, and rumination can be distinguished, based on their specific frequency patterns during grazing [7]. These approaches are especially suitable for detecting prehension events, which represent the fundamental unit of herbage intake and a key variable for estimating intake dynamics under field conditions. However, their application under practical grazing conditions, particularly in systems characterized by restricted grazing time and dynamic pasture depletion, remains limited.

Grazing behaviour is strongly influenced by temporal dynamics within the grazing session. As grazing progresses, pasture characteristics change due to progressive herbage depletion, which may affect bite mass and intake rate. It has been shown that herbage biomass declines rapidly during the initial phase of grazing and more gradually thereafter [8]. Grazing animals can adapt their ingestive behaviour to sward changes through compensatory mechanisms, typically increasing bite rate or changing locomotion behaviour when bite mass decreases [9,10]. However, the extent to which these mechanisms operate under conditions of high initial herbage availability and restricted grazing time remains unclear.

The aim of this study was to investigate the effects of within-session grazing dynamics on bite mass and herbage intake in dairy cows, combining acoustic detection of prehension events with direct observation of grazing behaviour.

## 2. Materials and Methods

### 2.1. Animals and Management

A total of 9 Pezzata Rossa lactating cows (initial milk production 7.5 ± 1.9 kg/day), were used in the experiment. Animals were managed under a pasture-based feeding system, receiving no supplementary feed during the grazing period. Each day, after the morning milking, cows were moved to pasture and allowed to graze for 5 h (0, 1, 2, 3 and 4), and were milked again approximately 12 h after the first milking. The trial lasted 2 months.

Grazing was conducted under a part-time grazing system, with animals allowed access to pasture for approximately 5 h per day.

Cows grazed on a mixed sward (mean botanical composition: 44% legumes, 40% grasses and 16% other species). On each of the four sampling dates, the nine experimental cows grazed for 5 h together with 19 additional cows from the same herd, resulting in a total group of 28 animals grazing within a single fenced area of 800 m^2^. A different fenced 800 m^2^ area within the same pasture was used on each sampling date, and each area was grazed only for 5 h.

Herbage allowance ranged from 16 to 19 kg DM per cow per day across the experimental dates, ensuring high initial herbage availability at pasture entry.

### 2.2. Data Collection

All measurements on pasture and animals were conducted on four sampling days at 15-day intervals over a 2-month period. On each day, measurements were taken at 0, 1, 2, 3, and 4 h of grazing.

Biomass was measured at each sampling time by cutting herbage at ground level using scissors within three 0.5 × 0.5 m quadrats randomly distributed within the grazed fenced area, and subsequently weighing the collected material in order to obtain representative estimates of pasture availability. Each herbage sample was manually separated into botanical components (legumes, grasses, and other species). Herbage samples from each quadrat, separated by botanical family, were collected for dry matter analysis [9]. Sward height was measured using a rigid meter at 20 random points across the grazed area, in order to account for spatial variability of the pasture.

Bite mass was estimated using the hand-plucking method. The technique consisted of closely observing the prehension modality of the grazing animal, identifying the specific plant parts severed, and manually replicating the observed defoliation pattern to collect an equivalent sample.

Preliminary repeatability assessments were conducted to evaluate the consistency and robustness of bite mass measurements under field conditions. Five trained observers, organized into two independent teams, simultaneously monitored grazing activity. Within each team, observers independently collected hand-plucked samples corresponding to prehension events from the same focal animal, without any communication or coordination between them, in order to avoid observer bias. Each observer collected 50 simulated bites, which were pooled into a single composite sample and weighed as one measurement to overcome the weight detection limits of individual bites and obtain a representative cumulative mass. This procedure allowed the assessment of inter-operator consistency under field conditions.

To evaluate the repeatability of the method, Dixon’s Q-test was applied to the pooled weights. The test identified one observer’s sample as a significant statistical outlier due to overestimation ($Q_{calculated}=0.652>Q_{critical}=0.642;p<0.05$). Conversely, the remaining four observers demonstrated a high level of consistency and cluster homogeneity. Therefore, once the overestimating operator was identified and excluded to avoid data distortion, two operators for the main experimental sessions were selected from within the homogeneous group of the remaining four.

At the beginning of each hour of grazing, bite mass was estimated within a short sampling window (~10 min), resulting in a total of 36 samples of 50 bites each across the four sampling days. A total of 40 hand-plucking samples was initially planned, with two replicate samples of 50 bites collected during each of the five grazing hours on each of the four sampling days. However, four incomplete samples collected under strong wind conditions on the final sampling day were excluded, resulting in a final dataset of 36 samples. Collected bite samples were then analyzed for dry matter content [11].

Individual milk yield was recorded at both daily milkings on each sampling day.

Grazing behaviour measurements were conducted by equipping each cow with a collar-mounted audio recorder (Olympus LS-P5, Olympus Corporation, Tokyo, Japan) and a point-of-view camera (GoPro Hero 11, GoPro, Inc., San Mateo, CA, USA), enabling the simultaneous collection of acoustic and visual data under field conditions.

**Figure 1:**
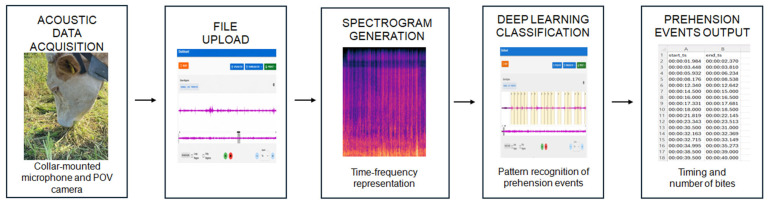
Workflow of the acoustic system for detecting herbage prehension events in grazing dairy cows, from data acquisition to deep learning–based classification and extraction of bite number and timing.

Audio recordings were processed using a custom-developed, password-protected web-based software tool [12], for which no formal version number is available. The software automatically detected prehension events and generated time-stamped CSV files (Figure 1).

Briefly, the acoustic tool processes audio signals by dividing them into non-overlapping 500-ms segments, transforming each segment into a logarithmic spectrogram, and classifying it as either prehension or no-prehension using a YOLO11s-cls deep-learning model developed with the Ultralytics framework (v8.3.174) and PyTorch (v2.4.1).

To ensure compatibility with the processing tool and to facilitate efficient data handling, the 5-h audio recordings were divided into 15-min segments. Of the 720 audio segments expected based on the experimental design, 596 were available for analysis due to occasional recording interruptions or technical issues, which are common under practical grazing conditions.

For each cow and sampling date, prehension events detected in consecutive 15-min recordings were summed to obtain one total prehension count for each grazing hour, thereby avoiding the treatment of consecutive recordings as independent observations. Pasture characteristics and bite mass were determined for each sampling date and grazing hour and were therefore common to all cows observed during the same hourly period. Accordingly, each row of the analytical dataset represented one cow × sampling date × grazing hour, combining the cow-specific hourly prehension count with the corresponding pasture and bite mass values for that sampling date and grazing hour.

For validation of the acoustic tool, 28 hourly observations were selected and evenly distributed across the experimental dates to ensure representative coverage of the experimental conditions. For each selected grazing hour, prehension counts from the four consecutive 15-min recordings were summed separately for the acoustic estimates and the corresponding manual counts, yielding 28 paired hourly observations.

### 2.3. Calculations

Hourly herbage intake was estimated as the product of the number of prehensions detected per hour and the corresponding estimated bite mass.

Intake over the entire grazing session was also independently calculated from biomass disappearance as the total reduction in herbage dry matter over the 800 m^2^ area divided by the 28 cows grazing within it. To evaluate the relationship between the two methods across the four sampling dates, the grazing session was divided into two consecutive intervals. Although pasture biomass was measured hourly, herbage intake based on biomass disappearance was calculated over two longer time intervals, corresponding to the first two grazing hours (hours 0 and 1) and the subsequent three grazing hours (hours 2, 3, and 4). Aggregating biomass disappearance over multi-hour periods was intended to reduce the influence of spatial sward heterogeneity and quadrat-sampling variability, which could have disproportionately affected estimates based on hourly biomass differences. For each sampling date and interval, individual acoustic-based intake estimates were summed over the corresponding hours and then averaged across the monitored cows. These mean values were paired with the corresponding estimates derived from biomass disappearance.

### 2.4. Statistical Analysis

A preliminary correlation analysis was conducted to explore the relationships between bite mass, number of prehensions per hour, dry matter intake per hour, and sward characteristics (biomass, herbage height, herbage dry matter content, and the percentage of legumes and grasses), with the aim of identifying potential associations among variables.

One-way ANOVA was used to evaluate the effect of grazing hour (0–4) on pasture characteristics (biomass, herbage height, and herbage dry matter content) and to assess the effect of sampling date on milk yield. Botanical composition variables, including the percentages of Fabaceae, Poaceae, and other botanical components, were also evaluated but were not further considered, as they did not show consistent or meaningful effects on the response variables.

A linear regression analysis was performed to evaluate the relationship, at the hourly level, between the number of prehensions estimated by the acoustic system (y) and those manually identified from video recordings (x), in order to quantify the agreement between the two measurement approaches. Precision was assessed using the coefficient of determination ($R^{2}$), whereas accuracy was evaluated by testing whether the regression intercept differed from zero and the slope differed from one.

Pearson’s correlation and linear regression were performed to evaluate the relationship between the biomass disappearance intake estimation and the individual acoustic based intake estimation on eight paired observations (4 dates over two time intervals, corresponding to the first two grazing hours and the subsequent three grazing hours).

Linear mixed models were used to evaluate the effect of grazing hour (0–4) on bite mass, number of prehensions, and herbage intake estimated through acoustic monitoring. Bite mass, number of prehensions, and herbage intake were included as dependent variables, with grazing hour as a fixed effect. Sampling date was included as a random effect in all models to account for temporal variability, while cow was additionally included as a random effect for the analysis of number of prehensions and herbage intake, in order to account for repeated measurements on the same individuals.

Statistical analyses were performed using Jamovi (version 2.6; The jamovi project, Sydney, Australia).

## 3. Results

The correlation analysis between bite mass and sward characteristics (biomass, herbage height, herbage dry matter content, percentage of legumes, and percentage of grasses) showed significant positive relationships only with biomass (r = 0.362, *p* = 0.046) and herbage dry matter content (r = 0.360, *p* = 0.047). No other significant correlations were found for bite mass.

The correlation matrix between the number of prehensions per hour, dry matter intake per hour, and sward characteristics is reported in Table 1. Significant relationships (*p* < 0.01) were observed between the two behavioural variables and most sward characteristics, except for the percentage of grasses, which was not significantly correlated.

**Figure 2:**
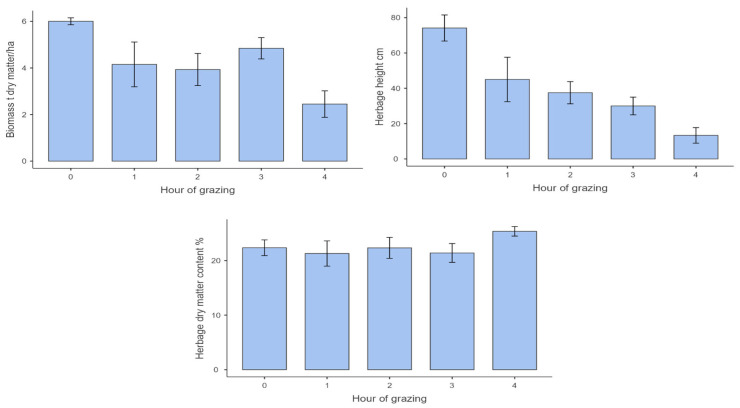
Changes in sward characteristics across grazing hours. Values are means ± standard error.

Figure 2 shows mean values of sward characteristics across grazing hours. A significant decrease in biomass (*p* = 0.025) and herbage height (*p* = 0.008) was found during the grazing day, whereas no significant effect was evident in herbage dry matter content (*p* = 0.326).

**Figure 3:**
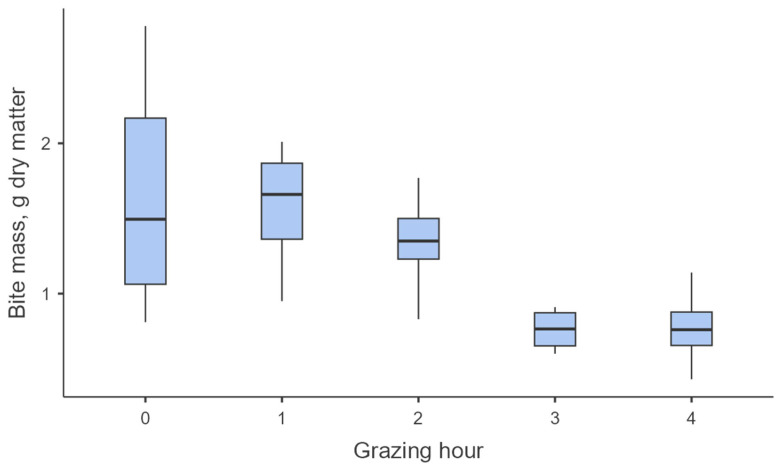
Variation in bite mass (g DM/bite) across grazing hours (0–4). Boxplots represent the distribution of observations within each grazing hour.

**Figure 4:**
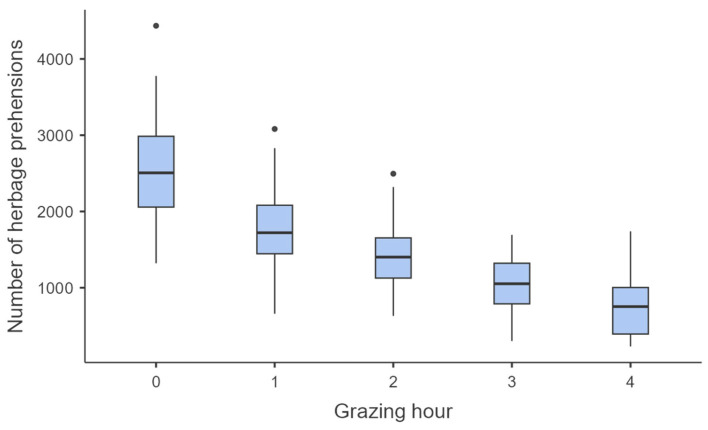
Variation in number of herbage prehensions across grazing hour (0–4) (n/h). Boxplots represent the distribution of observations within each grazing hour.

**Figure 5:**
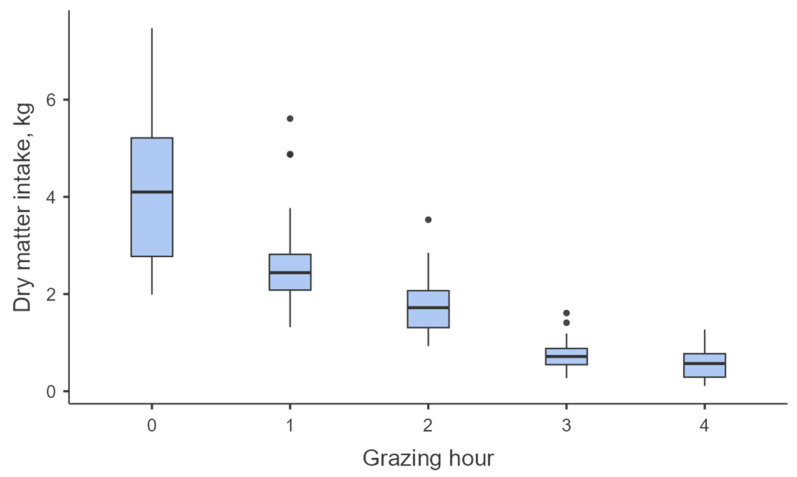
Variation in herbage dry matter intake (kg/h) across grazing hour (0–4). Boxplots represent the distribution of observations within each grazing hour.

Grazing hour significantly affected bite mass (*p* = 0.008), number of prehensions (*p* < 0.001), and hourly dry matter intake (*p* < 0.001). Bite mass decreased significantly from hour 3 onwards compared with hours 0, 1, and 2. The number of prehensions and hourly dry matter intake progressively decreased from hour 0 to hour 3, with no significant differences between hours 3 and 4 (Figure 3, Figure 4 and Figure 5).

**Figure 6:**
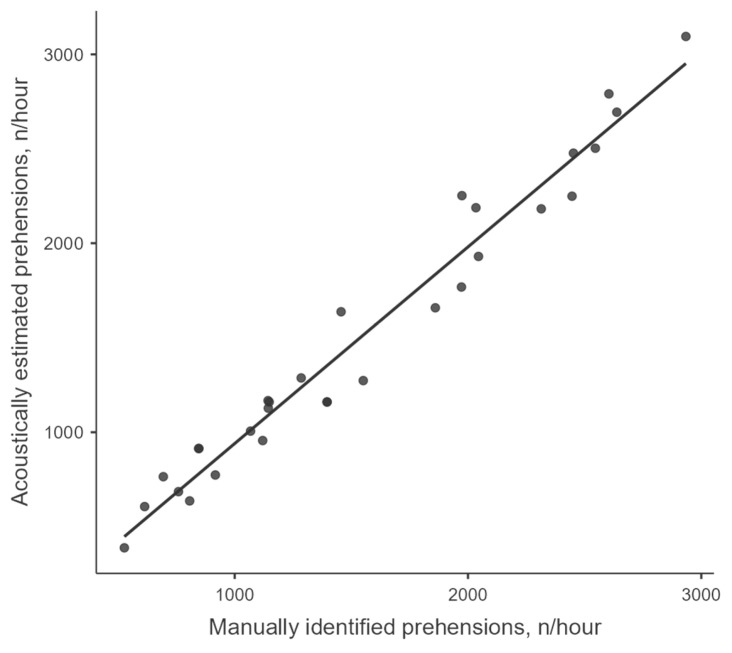
Relationship between the hourly number of prehensions estimated by the acoustic tool and that manually identified from video recordings. Each point represents an hourly count obtained by summing four consecutive 15-min recordings. The solid line represents the fitted linear regression. The regression equation was y=−99.81+1.04x, with $R^{2}=0.964$.

A strong relationship was observed between the hourly number of prehensions estimated by the acoustic tool and that manually identified from video recordings (n=28, $R^{2}=0.964$; Figure 6). The regression equation was y=−99.81+1.04x. The intercept did not differ significantly from zero (p=0.135), and the slope did not differ significantly from one (p=0.303), providing no evidence of significant systematic or proportional bias at the hourly level.

Daily herbage intake estimated from biomass disappearance was similar to that estimated using the acoustic approach (10.71 ± 1.84 vs. 10.50 ± 2.25 kg DM per cow, respectively; mean ± SD). Based on the eight paired estimates obtained for the first two and the subsequent three grazing hours across the four sampling dates, a positive correlation was observed between the two methods, although it did not reach statistical significance (r=0.699, p=0.054). Linear regression yielded a slope of 0.937, an intercept of 0.021, and an $R^{2}$ of 0.489.

Milk yield, averaging 7.54 ± 1.62 kg/head/day, did not show any significant variation during the two-month trial (*p* = 0.587).

## 4. Discussion

This study examines the role of grazing hour in shaping ingestive behaviour and evaluates the applicability of an acoustic system for the automatic detection of herbage prehensions under practical grazing conditions. The results indicate that acoustic monitoring can be effectively used to estimate the number of herbage prehensions under grazing conditions. The recording device was mounted on a lightweight, non-invasive collar that did not restrict the animals’ movements or interfere with grazing. The equipment was visually checked during the experimental sessions, and no displacement of the device, signs of discomfort, attempts to remove the collar, or alterations in normal grazing behaviour were observed.

At the hourly level, acoustically estimated and video-verified prehension counts were closely related, with the regression explaining 96.4% of the observed variability. Moreover, the regression intercept did not differ significantly from zero and the slope did not differ significantly from one, indicating that no statistically detectable systematic or proportional bias occurred under the present experimental conditions. Nevertheless, validation was based on 28 paired hourly observations, and further evaluation under a wider range of animals, sward conditions, and recording environments would strengthen the generalizability of these findings.

When combined with bite mass estimated through hand plucking, this approach enables the estimation of herbage intake and its temporal dynamics at the individual level.

Although the number of bite-mass observations was relatively limited, each sample represented the mean dry matter mass of 50 hand-plucked bites, corresponding to approximately 1800 individually collected bites across the entire experiment. This sampling procedure increased the robustness of the estimates and reduced the influence of variability among individual simulated bites.

Andriamandroso et al. [13], in a review of automatic systems for classifying jaw movements in grazing animals, highlighted that the prediction of herbage intake remains challenging, mainly due to the difficulty of accurately estimating bite mass. Although approaches based on structural parameters of the sward have been proposed, such as the relationship between bite mass, bite area, bite depth and bulk density [14], their practical application is limited under field conditions, particularly in heterogeneous pastures such as those investigated in the present study. In this context, hand-plucking remains one of the most reliable methods for estimating bite mass, with reported accuracy values of up to 95% [13].

The hand-plucking method has been criticized for its limited repeatability and for not fully representing the ingestive behaviour of grazing animals. Langlands [15], comparing hand-plucked samples with those collected from oesophageal-fistulated animals, reported that the former tended to overestimate the nutritive value of low-quality pastures.

However, the use of oesophageal-fistulated animals cannot be considered a definitive reference method, as it is affected by several sources of bias, including the short duration of sampling relative to the entire grazing period. This limitation is particularly relevant in heterogeneous pastures, where animals continuously modify their selective behaviour by changing feeding stations throughout the grazing session [16].

Bonnet et al. [17], in a study assessing the reliability of the method (hand plucking method), reported that trained observers can estimate bite mass with an accuracy exceeding 80%. Nevertheless, some degree of approximation cannot be excluded, as with any indirect method.

In the present study, two previously trained observers were involved in sample collection. In addition, a preliminary procedure was conducted to assess method repeatability, involving five trained observers organized into two independent teams, which simultaneously collected hand-plucked samples from the same focal animal without communication between them. This procedure was intended to assess the repeatability of the method and provides an indication of the reliability of bite mass measurements obtained in our conditions.

Video recordings obtained using point-of-view (POV) cameras were used to assess the validity of the acoustic tool in classifying prehension events. The use of POV cameras overcomes several limitations of traditional video recording, such as the distance from the animals, poor lighting conditions, and the potential disturbance caused by following grazing animals [18]. This approach has been proposed as one of the few methods capable of capturing the microstructure of grazing behaviour under field conditions, demonstrating its effectiveness in complex grazing environments [19].

The acoustic tool used, developed by our research team, is based on a deep learning approach capable of recognising spectrogram patterns associated with prehension events, achieving an accuracy of approximately 0.83 [12]. Previous studies on acoustic monitoring of grazing behaviour have often been conducted under highly controlled conditions, such as artificial grazing on microswards and short observation periods [20,21]. Nevertheless, several classification systems have shown promising performance [22].

The agreement observed between acoustic and video-based bite counts, although based on a subset of observations, indicates that the system performed consistently under the specific experimental conditions of the present study, which differ from those in which the tool was originally developed. Detailed performance metrics are reported in the original study describing the development and validation of the tool [12].

Mean daily herbage intake estimated from biomass disappearance was comparable to that obtained using the acoustic approach combined with hand-plucking measurements of bite mass (10.71 ± 1.84 and 10.50 ± 2.25 kg DM per cow, respectively). When the comparison was conducted using the eight paired estimates obtained over two consecutive grazing intervals across the four sampling dates, the two methods showed a positive association, although it did not reach statistical significance (r = 0.699, *p* = 0.054). The regression slope was close to one (0.937), whereas the intercept was close to zero (0.021), with an R^2^ of 0.489. These findings indicate consistency between the two approaches at the level of mean intake estimates, but the limited number of paired observations prevents firm conclusions regarding their agreement and accuracy. Further validation using a larger number of independent grazing sessions is therefore warranted.

Laca and WallisDeVries [23] suggested that chewing sounds may provide more information on intake than biting sounds or chews per bite. However, the highly controlled conditions of their study and the intrinsic complexity of chewing sound interpretation may limit the practical applicability of this approach under field conditions.

Pasture characteristics, as expected, showed significant relationships with behavioural variables. Bite mass was positively correlated with both herbage biomass and herbage dry matter content, whereas no significant relationship was observed with sward height. The positive association with biomass likely reflects the greater opportunity for animals to harvest larger bites when herbage availability is higher. Conversely, the positive relationship with herbage dry matter content should be interpreted mainly as a consequence of the calculation of bite mass on a dry matter basis, since dry matter concentration directly contributes to the estimated amount of DM per bite. No significant relationship between herbage height and bite mass was observed, despite the close correlation between herbage height and biomass. This may be explained by the high variability in sward height, which could have reduced the statistical power of the analysis.

The number of prehensions showed significant correlations with most sward characteristics, except for the proportion of grasses. However, these relationships should be interpreted with caution, as grazing hour is inherently associated with changes in pasture characteristics. To account for this potential confounding effect, the same correlation analysis was performed considering only data from grazing hour 0. Under these conditions, the only parameter significantly associated with the number of prehensions was herbage dry matter content, which showed a negative relationship, possibly reflecting increased difficulty of grazing as forage quality declines.

A progressive decrease in both bite mass and number of prehension bites was observed from the beginning to the end of the grazing session. This pattern is consistent with previous evidence showing that bite mass and intake rate may be greater during the early phase of a grazing bout and decline as grazing progresses. Chilibroste et al. [24] and Alvarez-Hess et al. [4] reported higher bite mass and number of prehension bites during the first hour of grazing than later in the grazing session. However, these results should not be interpreted as a simple effect of pre-grazing herbage mass. Alvarez-Hess et al. [4] found no significant effect of herbage mass on daily bite mass, whereas bite mass increased with greater herbage allowance per head per day. Similarly, Rombach et al. [25], comparing paddocks with markedly different pre-grazing herbage mass but the same target daily herbage allowance, reported similar bite mass and number of prehension bites between treatments. Taken together, these findings suggest that, under the experimental conditions of the present study, the reduction in bite mass and number of prehension bites over the grazing session was likely driven by the combined effects of within-session pasture depletion and progressive satiation, rather than by initial herbage biomass alone. As grazing progressed, pasture biomass declined, contributing to a progressive reduction in accessible herbage per cow during the grazing session, despite the relatively high daily herbage allowance. Under the constant stocking rate applied throughout the experiment, herbage allowance was directly related to pasture biomass.

It is widely reported that grazing animals respond to reduced pasture availability—either in terms of biomass or access time—by adopting compensatory behavioural mechanisms aimed at maintaining intake. These responses may include an increase in biting rate or a reduction in non-feeding activities such as idling and rumination [26,27,28,29]. However, this compensatory grazing behaviour, typically characterized by an increase in bite number under reduced herbage availability, was not observed in the present study. This may indicate that cows achieved a relatively high intake rate during the initial grazing hours, thereby reducing the need or the physiological drive for compensatory grazing behaviour later in the session. This interpretation is consistent with previous findings showing that rumen fill plays a key role in regulating ingestive behaviour, progressively reducing intake rate and feeding activity as satiety develops [30]. Similar responses have been reported in previous studies on sheep [31], where reducing grazing time from 7 to 4 h, under moderate temperature conditions, did not reduce herbage intake and was even associated with increased milk production. Those findings suggest that animals may adapt their behaviour to restricted grazing time, potentially learning to optimize intake within a limited time window.

## 5. Conclusions

Overall, these results indicate that, despite the inherent variability of individual intake components, the combined use of acoustic detection of prehension events and bite mass estimation through hand plucking provides a practical and informative approach for estimating total daily herbage intake. Importantly, this method also enables the assessment of individual ingestive behaviour and its temporal dynamics during the grazing session. Furthermore, while biomass-based methods remain simpler and widely applicable at the group level, the proposed approach allows intake estimation at the individual level and provides a more detailed characterization of ingestive processes under grazing conditions. The application of this method made it possible to highlight a progressive decrease in both bite mass and number of prehension bites from the beginning to the end of the short grazing session. This behavioural response to grazing hour was likely driven by the combined effects of within-session pasture depletion and progressive satiation.

## Figures and Tables

**Table 1:** Correlation coefficient (r) values and *p* values (in brackets) between herbage prehensions (n/h) and intake, (kg DM ^(1)^/h) with pasture characteristics.

	Herbage Prehensions n/h	DM ^(1)^ Intake kg/h
Herbage prehensions, n/h	-	0.850
	-	(<0.001)
DM intake, kg/h	0.850	-
	(<0.001)	-
Biomass, t DM ^(1)^/ha	0.392	0.472
	(<0.001)	(<0.001)
Herbage height, cm	0.631	0.520
	(<0.001)	(<0.001)
Herbage DM ^(1)^ content, %	−0.248	0.048
	(0.005)	(0.592)
Legumes, % DM ^(1)^	0.247	0.331
	(0.003)	(<0.001)
Grasses, % DM ^(1)^	−0.159	−0.239
	(0.061)	(0.005)

^(1)^ DM, dry matter.

## Data Availability

The data presented in this study are available on reasonable request from the corresponding author. Raw audio/video recordings and animal-level datasets are not publicly archived due to their large file size and institutional data-storage limitations.
